# Resistive Switching Characteristics of Alloyed AlSiO_x_ Insulator for Neuromorphic Devices

**DOI:** 10.3390/ma15217520

**Published:** 2022-10-26

**Authors:** Yunseok Lee, Jiung Jang, Beomki Jeon, Kisong Lee, Daewon Chung, Sungjun Kim

**Affiliations:** 1 Division of Electronics and Electrical Engineering, Dongguk University, Seoul 04620, Korea; 2 Department of Information and Communication Engineering, Dongguk University, Seoul 04620, Korea

**Keywords:** neuromorphic system, memristor, resistive switching, alloyed materials

## Abstract

Charge-based memories, such as NAND flash and dynamic random-access memory (DRAM), have reached scaling limits and various next-generation memories are being studied to overcome their issues. Resistive random-access memory (RRAM) has advantages in structural scalability and long retention characteristics, and thus has been studied as a next-generation memory application and neuromorphic system area. In this paper, AlSiO_x_, which was used as an alloyed insulator, was used to secure stable switching. We demonstrate synaptic characteristics, as well as the basic resistive switching characteristics with multi-level cells (MLC) by applying the DC sweep and pulses. Conduction mechanism analysis for resistive switching characteristics was conducted to understand the resistive switching properties of the device. MLC, retention, and endurance are evaluated and potentiation/depression curves are mimicked for a neuromorphic device.

## 1. Introduction

Recently, the exponential growth of information and data related to AI and big data requires higher computing performance than before. The existing von Neumann structure cannot meet high-performance computing due to its serial processing of information. Therefore, neuromorphic computing, which mimics the human brain with parallel processing is more suitable for better energy efficiency and performance processing [1,2,3,4]. To implement the neuromorphic system, the parallel structures in hardware that can provide multi-level states stored in memory elements by vector–matrix products are required [5]. RRAM with a metal–insulator–metal configuration is a suitable crossbar array memory structure for parallel processing. The memory characteristics in RRAM are achieved by altering the resistance value of the insulator layer [6,7,8,9]. RRAM has several advantages of a simple structure, long retention, fast switching, and MLC for neuromorphic systems [10,11,12,13,14,15,16].

The scaling issues of Si-based transistors, such as the short channel effect, induced research on various gate dielectrics, such as HfO_2_ and Al_2_O_3_, in addition to SiO_2_. Recently, Hf-, Zr-, and Al-based oxides have become candidates for future high-k materials, having relatively large band gaps and good compatibility with Si. Because of their high dielectric constant, stability with Si, and relatively large band gap, Hf-, Zr-, and Al-based oxides have recently emerged as candidates for various semiconductor applications [17,18]. SiO_2_ as a conventional gate insulator has a band gap value of ~9.0 eV and a dielectric constant value of 3.9. Aluminum oxide (Al_2_O_3_) has a similar band gap to SiO_2_, and it has a dielectric constant of about 9, which is at least two times that of SiO_2_ [19,20]. In particular, the Al_2_O_3_ film with its good insulation properties can be a good material for controlling the resistance properties. High-k composite semiconductor technology has recently been adopted in various ways, such as a stacked bilayer structure or an alloy structure, taking advantage of the benefits of each dielectric, such as the excellent interface quality of Al_2_O_3_ and the accumulated research reports of SiO_2_ [21,22]. Table 1 compares various parameters for related previous studies fabricated RRAM devices composed of alloyed and bilayer types [23,24,25,26,27]. This paper investigates the resistive switching characteristics of the dielectric in the form of an alloy with two effective insulating layers.

In this paper, an alloyed-type RRAM that switches using an insulator layer produced by alternately deposing Al and Si layers by an atomic layer deposition (ALD) process was investigated. The device is composed of Pt/Ti/AlSiO_x_/W. The conduction mechanism in the device is investigated using I-V fitting, as well as basic I-V and MLC characteristics are demonstrated. Furthermore, the endurance characteristics are confirmed by pulse measurement, and each state could be gradually controlled by potentiation and depression for the implantation of neuromorphic computing.

## 2. Experiments

The fabrication process of the Pt/Ti/AlSiO_x_/W device is described as follows. Sputtering was used to deposit a 100 nm thick W on a SiO_2_/Si substrate wafer. Following that, an ALD method was employed to deposit a 5 nm thick AlSiO_x_ as an insulator. The deposition was alternately carried out with AlO_x_ and SiO_x_ layers. Trimethylaluminurn (TMA) was used as an Al_2_O_3_ precursor and diisopropylaminosilane (DIPAS) was used as a SiO_2_ precursor for the alloyed layer. Ti was deposited as an adhesion layer on the AlSiO_x_ layer before Pt deposition as the top electrode (TE). Once the deposition was completed, a 100 nm thick Pt was then deposited on the Ti layer. The area between each cell was defined using a shadow mask during TE deposition. Keithley’s 4200-SCS and 4225-PMU semiconductor parameter module machines were used to measure electrical properties for this work. All measurements were carried out by controlling TE bias and fixing bottom electrode (BE) as ground.

## 3. Results and Discussions

**Figure 1:**
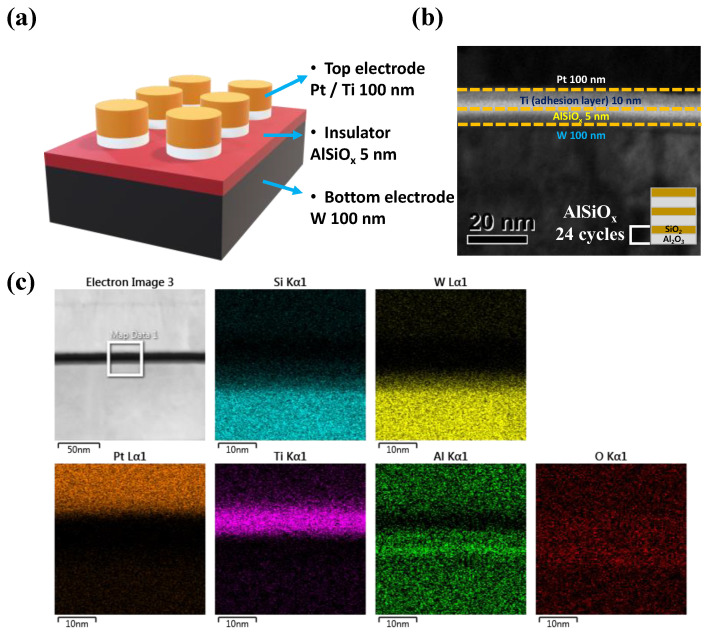
(**a**) Schematics image of Pt/Ti/AlSiO_x_/W device, (**b**) cross-sectional TEM image, (**c**) EDS mapping images of Si, W, Pt, Ti, Al, and O elements collected from the area indicated in the TEM image of the Pt/Ti/AlSiO_x_/W device.

The schematic of the device used in this paper is shown in Figure 1a. The device is composed of a metal–insulator–metal structure, the same as the element configuration of RRAM, with Pt/Ti for TE, AlSiO_x_ for insulator, and W for BE. Furthermore, through the transmission electron microscopy (TEM) image in Figure 1b, it is possible to verify the device stack. The deposition method of the AlSiO_x_ insulator layer is depicted in the inset of Figure 1b. The energy dispersive X-ray spectroscopy (EDS) mapping for each layer is shown in Figure 1c. Note that it is proven that the Al and Si element distribution is uniformly deposited in the AlSiO_x_ insulator layer.

**Figure 2:**
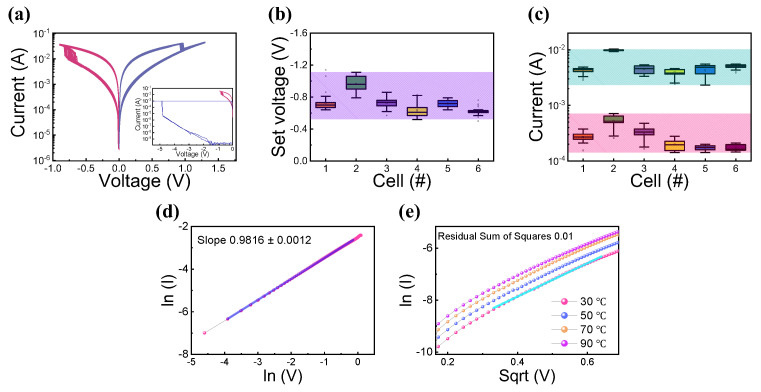
(**a**) Bipolar resistive switching of Pt/Ti/AlSiO_x_/W device. Box charts for cell-to-cell variation: (**b**) set voltage, (**c**) HRS and LRS at reading voltage of 0.1 V. Conduction mechanism I-V fitting: (**d**) in LRS, (**e**) in HRS.

All DC sweep measurement is conducted by increasing or decreasing the step voltage of 0.01 V in Figure 2a–e. The typical I-V characteristics of the device are shown in Figure 2a. In the inset Figure 2a, the compliance current of 1 mA was used to prevent excessive filament growth from the initial state. The set and reset voltages were set to approximately −0.8 V and −1.2 V. The compliance current was not applied to the device due to the self-compliance behavior. This indicates that the resistance values of the device can be controlled by voltage. The electrodes of the device were made of non-diffusion metals with W and Pt. As a result, an oxygen vacancies-based filament in the insulators is formed by the voltage bias [28,29]. Cycle-to-cycle variability is affected by the number of oxygen vacancy defects that arise in the stochastic nature of conductance filament formation and rupture during resistive switching. A device with a large cell area lacks uniformity in the various device characteristics [30,31]. The set voltage and current level uniformity were confirmed by switching during 30 cycles. The box plot of set voltage for each cell is shown in Figure 2b, with a variation of less than 0.4 V. A uniform current level is observed in the high-resistance state (HRS) and low-resistance state (LRS) with on/off ratio of about 10, in Figure 2c. Although the device has a pretty good variation, it has the disadvantage of high current level. According to previous studies, if the CC is set low, the operating current can be lowered, but it has the disadvantage of increasing the dispersion of HRS and LRS [32]. In addition, effective results have been reported for reducing the current by reducing the area of the device [33]. The conduction mechanism was confirmed using the I-V fitting during the device’s operation. In Figure 2d, $\ln\left(V\right)$ vs. $\ln\left(I\right)$ indicates that the electron transport mechanism follows ohmic conduction in LRS. This proves that the current flows through the strong conducting filament formed by defects in the insulator layer [34,35,36]. As shown in Figure 2e, the linear fitting of √V vs. $\ln\left(I\right)$ is well matched with measured I-V curves in HRS. This indicates the electron transport mechanism could be Schottky emission in HRS. Moreover, similar characteristics are observed at a higher temperatures from 30 °C to 90 °C [35,36,37,38].

**Figure 3:**
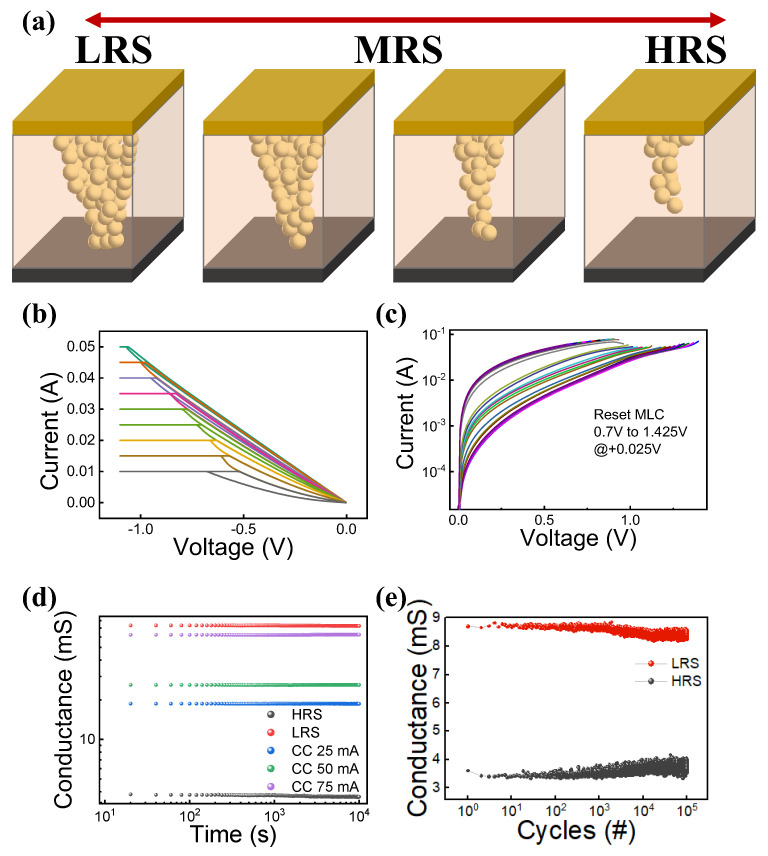
(**a**) Schematic diagram of the filament for each state. MLC characteristics: (**b**) in set region, (**c**) in reset region. (**d**) Multi-states retention characteristics for 10^4^ s, (**e**) endurance characteristic of the device for 10^5^ cycles.

The ability of the device to implement multi-states in both HRS and LRS has an advantage in terms of low-cost and high-density non-volatile data storage solution. Figure 3a depicts a schematic diagram for the filament transformation. The size of the filament could affect the resistance state of the device. By altering the size of the filament, various middle resistance states (MRS) may be achieved in addition to HRS and LRS. It can be implemented with techniques, such as controlling the reset voltage in the reset operation and limiting the compliance current in the set operation [39,40,41,42]. Figure 3b,c depict the MLC characteristic that can be implemented in the DC switching process. Figure 3b shows nine different states from 10 mA to 50 mA by increasing compliance current. In Figure 3c, the MLC characteristic with more states could be performed by a very small voltage change even during the reset process. The retention and MLC characteristics and endurance characteristics are representative elements of RRAM performance metrics [33]. Figure 3d demonstrates each state’s retention capacity. Current levels were shown differently depending on compliance current and a retention test that was performed for each state. As a result, the data retention of the device could be confirmed at not only the HRS and LRS but also the MRS by controlling compliance current of 25, 50, and 75 mA. It is defined as the number of trusted on/off behavior switching times in HRS and LRS. This device’s endurance characteristics are depicted in Figure 3e. The set and reset cycle process was performed more than 10^5^ times by pulses. Despite a large number of cycles, there is little variation in the conductance of the HRS and LRS.

**Figure 4:**
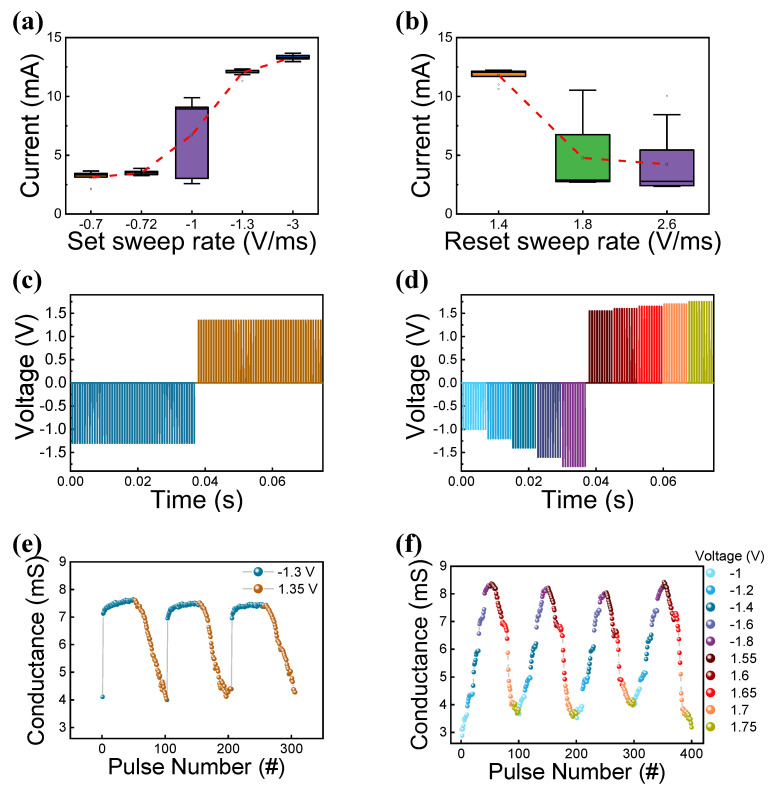
Current analysis by sweep rate V/t control: (**a**) LRS, (**b**) HRS. Potentiation and depression input voltage graph: (**c**) identical pulse segment type, (**d**) incremental pulse segment type. Potentiation and depression output conductance graph: (**e**) identical pulse segment type, (**f**) incremental pulse segment type.

The sweep rates of the set and reset operations are shown in Figure 4a,b. The device exhibits sweep rate-dependent resistive switching for the same pulse time. In Figure 4a, as the set sweep rate increases, higher voltage amplitude is applied within the same pulse width. From HRS, a small current change in the range of −0.7 V/ms to −1 V/ms was observed, but a large current change in the range of −1 V/ms to −3 V/ms indicates that the device reaches the LRS. Figure 4b shows the current change with the reset sweep rate. The device was not significantly changed from the LRS at the sweep rate of 1.4 V/ms, but the devices turns to HRS at 1.8 V/ms and 2.6 V/ms. In both Figure 4a,b, the red dotted line connects the mean value exhibiting the tendency of the amount of change to increase as a function of the amount of sweep rate. Potentiation and depression are the changes in synaptic strength, induced by specific patterns of synaptic activity, that have received much attention as cellular models of information storage in the central nervous system [43]. Conductance changes in devices by pulse can mimic synaptic characteristics in order to relate to the neuromorphic characteristics that are intended to be implemented similarly to human brain structures [44]. Figure 4c,d show the input voltage by identical pulse segment type and the incremental pulse segment type, respectively. The programming pulse time was 50 μs. The voltage for each segment is specified in Figure 4e,f. For potentiation and depression, a continuous programming pulse was applied 50 times each. The resulting change in conductance of the device can be seen in Figure 4e,f, respectively. As shown in Figure 4e, the potentiation and depression by the identical pulses have a range of about 4 mS to 7.5 mS, and it can be seen that it is particularly abrupt in the potentiation part. On the other hand, the pulse measurement result shows a more gradual conductance change by using an incremental pulse in Figure 4f [45,46]. In addition, conductance changes can be proven across a wider range of approximately 3 mS to 8.5 mS. The implementation of various synaptic weights through improved gradualness has the advantage of synaptic characteristics and, furthermore, improves the accuracy performance in the simulations, such as MNIST’s handwritten digit recognition [47].

## 4. Conclusions

In summary, the Pt/Ti/AlSiO_x_/W device was prepared by sputtering and ALD. Electrical characteristics of the alloyed AlSiO_x_-based device were conducted by DC sweep and pulse. The configuration of the device is confirmed by TEM and EDS analysis. The typical resistive switching was characterized and the conduction mechanisms of each state were analyzed. The uniformity, retention, and endurance prove the suitability of the device as a memristor. MLC characteristics were conducted by controlling compliance current and reset voltages. The I-Vs^−1^ analysis shows the degree of state change by pulse amplitude. The synaptic properties were validated with different pulse conditions. In conclusion, the alloyed AlSiO_x_-based device’s resistive characteristics and suitability for neuromorphic computing were demonstrated.

## Figures and Tables

**Table 1:** Comparison of alloyed and bilayer type RRAM in terms of memory device characteristics.

Device Structure	Set Voltage (V)	Reset Voltage (V)	Current Level	On–Off Ratio	Retention (s)	MLC	Synaptic Characteristics
ITO/HfAlO/TaN–NP/HfAlO/ITO	−0.7	1	10 μA–1 mA	>10	>10^4^	O	O
TiN/Ti/HfAlO/ITO	−0.5	0.75	10 μA–100 μA	>10	>10^4^	O	O
Au/Ti/HfTiO_x_/p-Si	6	−2.5	1 nA–100 μA	>10^4^	>10^4^	-	O
Pt/HfAlO_x_/TiN	−1.5	1.5	10 μA–100 μA	>10	-	O	O
Ag/FeZnO/Pt	0.75	−1	10 μA–10 mA	>3.8 × 10^2^	>10^7^	-	-
Ag/FeZnO/MgO/Pt	1.5	−1	10 nA–10 mA	>9.9 × 10^5^	>10^7^	-	-
Pt/Ti/AlSiO_x_/W	−0.8	1.2	100 μA–5 mA	>10	>10^4^	O	O

## Data Availability

Not applicable.
